# Supplementary material for: Reconciling Mining with the Conservation of Cave Biodiversity: A Quantitative Baseline to Help Establish Conservation Priorities
Source: PLoS One. 2016 Dec 20;11(12):e0168348. doi: 10.1371/journal.pone.0168348 (PMC5173368; doi:10.1371/journal.pone.0168348)
Supplement: S1 Dataset — (ZIP) [file pone.0168348.s002.zip › Taxa/Serra Norte/SN_2013/N4E/Lista N4E-59.pdf]

CAVIDADE N4E-0059

Classe	Ordem	Fam/Outros	Gên/Outros	Espécie	S-U	U-S	Zon.
Annelida	Oligochaeta			jovem		1	E
Arachnida	Acari	Bdellidae		sp.2		X	E
Arachnida	Amblypygi	Phrynidae	*Heterophrynus*	*longicornis*	2		E
Arachnida	Araneae	Ctenidae		jovem	2	2	E
Arachnida	Araneae	Ochyroceratidae		jovem		X	E
Arachnida	Araneae	Pholcidae	*Leptopholcus*	sp.1	X	X	E
Arachnida	Araneae	Pholcidae	*Mesabolivar*	sp.1	X	X	E
Arachnida	Araneae	Salticidae		jovem		X	E
Arachnida	Araneae	Scytodidae	*Scytodes*	*eleonorae*		2	E
Arachnida	Araneae	Theridiidae		jovem		X	E
Arachnida	Araneae	Theridiosomatidae	*Plato*	sp.1		X	E
Arachnida	Opiliones	Cosmetidae	*Roquettea*	*singularis*		1	E
Arachnida	Opiliones	Stygnidae	*Protimesius*	*gracilis*	1		E
Arachnida	Pseudoscorpiones	Chernetidae	*Spelaeochernes*	sp.1	X		E
Arachnida	Ricinulei	Ricinoididae	*Cryptocellus*	jovem		1	E
Chilopoda	Scutigeromorpha			sp.		1	E
Diplopoda	Glomeridesmida	Glomeridesmidae		sp.1		2	E
Diplopoda	Polydesmida	Chelodesmidae		sp.4		1	E
Diplopoda	Polydesmida	Paradoxosomatidae		sp.1		2	E
Entognatha	Collembola	Paronellidae		sp.1		X	E
Entognatha	Diplura	Campodeidae		sp.1	X	X	E
Insecta	Blattodea			jovem	2		E
Insecta	Coleoptera	Scydmaenidae		sp.3	X	X	E
Insecta	Dermaptera	Anisolabididae		sp.1		1	E
Insecta	Diptera	Psychodidae	Phlebotominae	sp.		X	E
Insecta	Diptera	Tipulidae	Tipulinae	sp.	X		E
Insecta	Heteroptera	Reduviidae	Emesinae	sp.6		1	E
Insecta	Homoptera	Cixiidae		jovem	X		E
Insecta	Hymenoptera	Formicidae	Myrmicinae	sp.1		X	E
Insecta	Hymenoptera	Formicidae	*Odontomachus*	sp.2		X	E
Insecta	Hymenoptera	Formicidae	*Pachycondyla*	sp.1		X	E
Insecta	Hymenoptera	Formicidae	*Pachycondyla*	sp.2		X	E
Insecta	Hymenoptera	Formicidae	*Paratrechina*	sp.1		X	E
Insecta	Hymenoptera	Formicidae	*Paratrechina*	sp.2	X		E
Insecta	Hymenoptera	Formicidae	*Pheidole*	sp.2	X	X	E
Insecta	Hymenoptera	Formicidae	*Trachymyrmex*	sp.1		X	E
Insecta	Isoptera	Termitidae	*Nasutitermes*	sp.	X	X	E
Insecta	Lepidoptera	Noctuoidea		sp.5		2	E
Insecta	Orthoptera	Gryllidae		jovem	1		E
Insecta	Orthoptera	Phalangopsidae	*Paraclodes*	sp.			E
Insecta	Orthoptera	Phalangopsidae	*Phalangopsis*	sp.	3	2	E
Insecta	Psocoptera	Epipsocidae	*Epipsocus*	sp.2	X		E
Gastropoda	Pulmonata	Bulimulidae	*Naesiotus*	sp.		X	E
Gastropoda	Pulmonata	Subulinidae		sp.3		X	E
Gastropoda	Pulmonata	Systrophiidae	*Happia*	sp.	X		E
Malacostraca	Isopoda	Philosciidae		sp.2		X	E
Amphibia	Anura	Strabomantidae	*Pristimantis*	cf. *fenestratus*	12		E
Mammalia	Chiroptera	Emballonuridae	*Peropteryx*	*kappleri*	2	3	E
